# The Role of the Proteinase Inhibitor Ovorubin in Apple Snail Eggs Resembles Plant Embryo Defense against Predation

**DOI:** 10.1371/journal.pone.0015059

**Published:** 2010-12-03

**Authors:** Marcos Sebastián Dreon, Santiago Ituarte, Horacio Heras

**Affiliations:** Instituto de Investigaciones Bioquímicas de La Plata (INIBIOLP), CONICET-Universidad Nacional de La Plata, La Plata, Argentina; Griffith University, Australia

## Abstract

**Background:**

Fieldwork has thoroughly established that most eggs are intensely predated. Among the few exceptions are the aerial egg clutches from the aquatic snail *Pomacea canaliculata* which have virtually no predators. Its defenses are advertised by the pigmented ovorubin perivitellin providing a conspicuous reddish coloration. The nature of the defense however, was not clear, except for a screening for defenses that identified a neurotoxic perivitellin with lethal effect on rodents.

Ovorubin is a proteinase inhibitor (PI) whose role to protect against pathogens was taken for granted, according to the prevailing assumption. Through biochemical, biophysical and feeding experiments we studied the proteinase inhibitor function of ovorubin in egg defenses.

**Methodology/Principal Findings:**

Mass spectrometry sequencing indicated ovorubin belongs to the Kunitz-type serine proteinase inhibitor family. It specifically binds trypsin as determined by small angle X-ray scattering (SAXS) and cross-linking studies but, in contrast to the classical assumption, it does not prevent bacterial growth. Ovorubin was found extremely resistant to *in vitro* gastrointestinal proteolysis. Moreover feeding studies showed that ovorubin ingestion diminishes growth rate in rats indicating that this highly stable PI is capable of surviving passage through the gastrointestinal tract in a biologically active form.

**Conclusions:**

To our knowledge, this is the first direct evidence of the interaction of an egg PI with a digestive protease of potential predators, limiting predator's ability to digest egg nutrients. This role has not been reported in the animal kingdom but it is similar to plant defenses against herbivory. Further, this would be the only defense model with no trade-offs between conspicuousness and noxiousness by encoding into the same molecule both the aposematic warning signal and an antinutritive/antidigestive defense. These defenses, combined with a neurotoxin and probably unpalatable factors would explain the near absence of predators, opening new perspectives in the study of the evolution and ecology of egg defensive strategies.

## Introduction

Decades of fieldwork have thoroughly established that the eggs of most animals are subject to intense predation [1]–[3]. The reason is clear: Their high nutritional value offers to a pest or pathogen the best target for attack [4].

**Figure 1:**
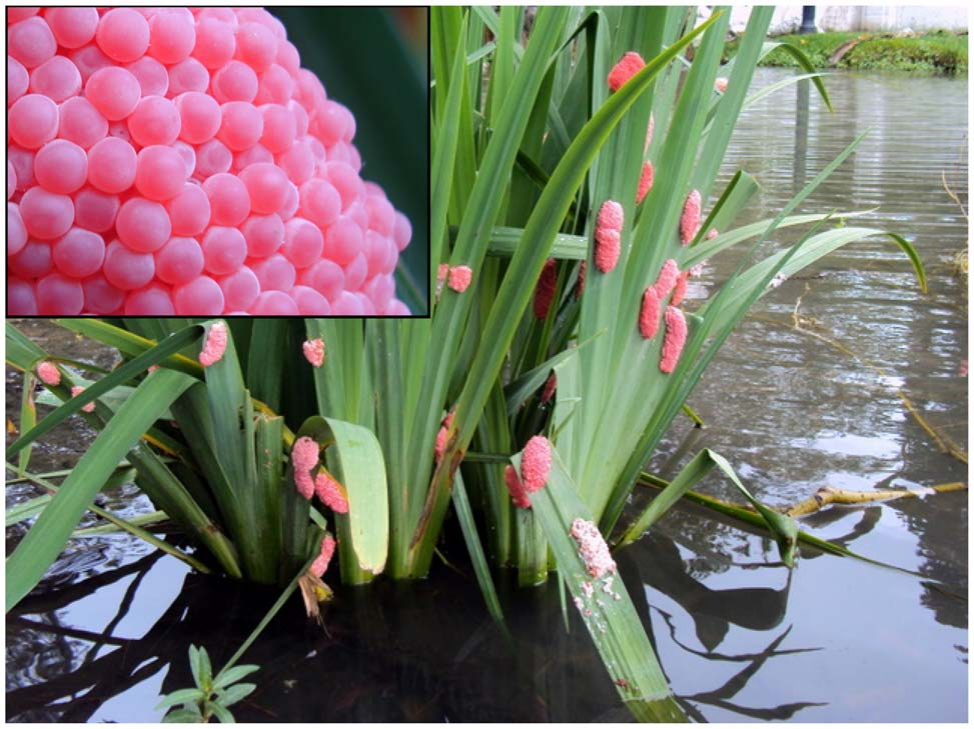
The conspicuous reddish egg clutches from *P.canaliculata* display a warning signal mostly provided by the perivitellin ovorubin. Inset: Egg surface does not have any protective ornamentation.

Among the few exceptions are the eggs from the freshwater apple snail *Pomacea canaliculata* which, though filled with large amounts of polysaccharides and proteins [5], have only one predator reported worldwide: the fire ant *Solenopsis geminata* [6]. *P. canaliculata* egg clutches are unusual in two respects: they are cemented outside the water and they are brightly coloured [7]–[9]. The strategy of laying eggs off the water allows eggs from aquatic organisms to avoid aquatic predators but at the same time they must face a variety of selective challenges, since they are exposed to stressful environmental conditions that may affect embryonic development and survival of offspring [10]; [11]. On the other hand, the conspicuously reddish coloration of the clutches (Figure 1) [12] advertises to visual-hunting predators the presence of egg defenses (aposematic warning). The message says: avoid me or pay the costs of a very unpleasant and/or unprofitable experience. However, the nature of these defenses remained a mystery until recently when, searching for defenses against predation, our group identified and characterized a proteinaceous neurotoxin (PV2) lethal to mice, the first genetically encoded toxin located inside an egg in the animal kingdom [13]; [14]. Eggs are toxic if orally administered to mice, but this slow-acting neurotoxin alone could not account for the virtual absence of predators, strongly suggesting the presence of other complementary noxious and/or unpalatable defensive factors, as the potential unpalatability reported for the eggs of a related species *P. paludosa* [15]. As in most gastropods, the female albumen gland provides eggs with the perivitellin fluid surrounding the fertilized oocyte to nourish and protect the embryos. Perivitellin fluid proteins, called perivitellins, have classically been considered merely storage proteins but recent work has shown that many of them serve other functions before being ingested by the embryos. For instance they provide eggs with lectins, proteinase inhibitors and other antimicrobial agents [16]–[20], growth factors for the developing embryo [21] and, in the case of *P. canaliculata*, a neurotoxin [13].

In particular, the presence of proteinase inhibitors in eggs, has classically been assumed to play a role either to protect against microbial infection (inhibiting extracellular proteases secreted by microorganisms) [4] or to minimize degradation of important peptides and proteins from egg vitellus or perivitellus [22]. However, despite its intuitive appeal, the antimicrobial hypothesis has been proved only in egg PIs of very few species, such as the eggs of the amphibian, *Odorrana grahami* [23].

*P. canaliculata* eggs have a perivitellin called ovorubin which is a strong proteinase inhibitor [24], that is at the same time pigmented with a carotenoid, providing eggs with their aposematic coloration. This multifunctional protein is massively accumulated in the perivitellin fluid [25], providing protection against sun radiation [12], stabilizing and transporting antioxidant molecules in the perivitellin fluid [26] and helping to prevent egg dessication [27]. As in other eggs, ovorubin PI function was assumed to be antimicrobial based on its capacity to inhibit *in vitro* the bacterial proteinases subtilisin and fungal takadiastase but this hypothesis has never been tested [24].

Several structural features of this 300 kDa oligomeric perivitellin have been studied and relevant for the current work are its high stability in a wide range of pH and temperature and elevated glycosylation [28]–[30].

In the present study we investigated some structural and functional aspects of ovorubin as proteinase inhibitor in *P. canaliculata* egg defenses through a combination of biochemical, biophysical and feeding experiments. First we studied the primary structure of ovorubin and its interaction with trypspin. Then we tested if the proteinase inhibitor properties of ovorubin conform the “antimicrobial assumption” and provide evidence that it is an antinutritive factor with a role in egg biochemical defenses that would render them unprofitable for a predator.

## Results

### Mass spectrometry analysis and sequencing

As a first step, we conducted a structural analysis of ovorubin studying its primary structure by mass spectrometry and Edman degradation. This led to the inclusion of ovorubin into the family of Kunitz-type serine proteinase inhibitors.

**Figure 2:**
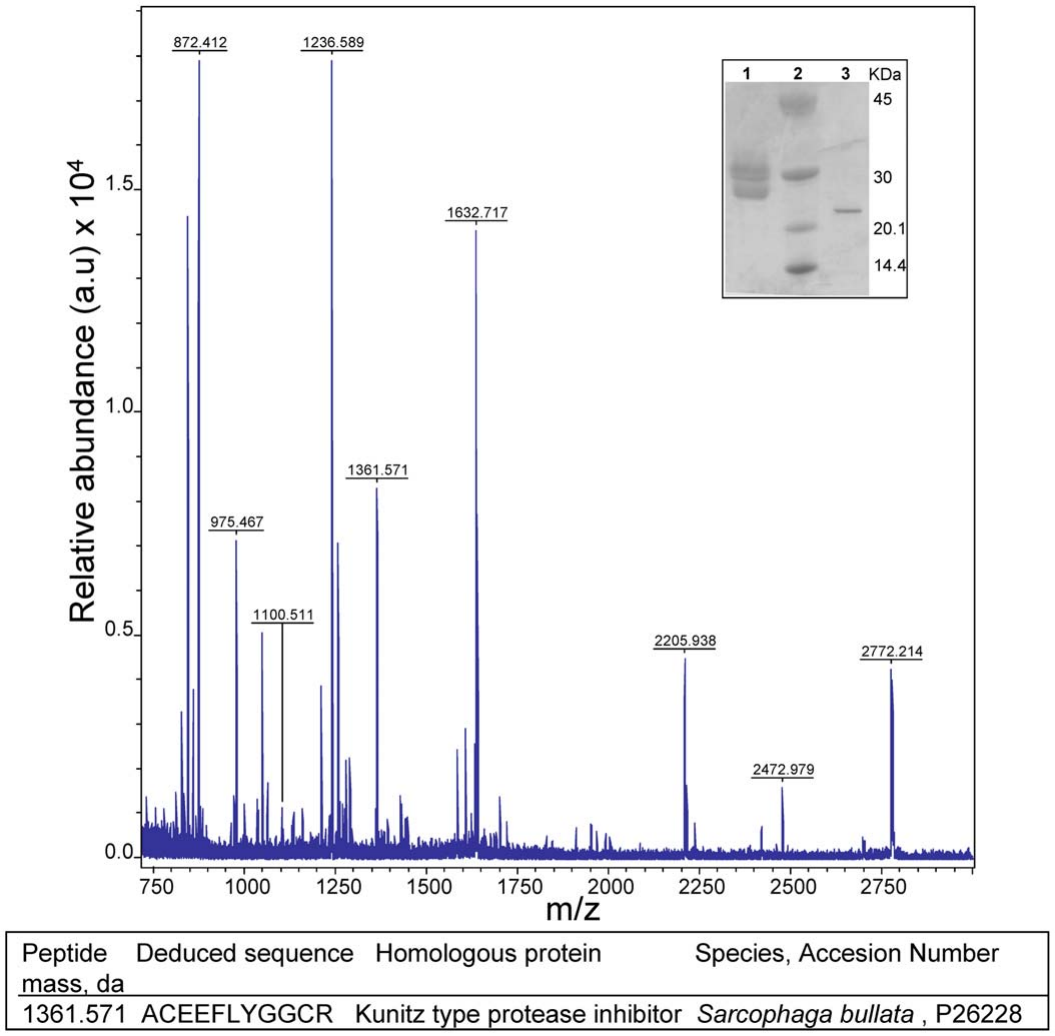
Tryptic digest fingerprint of deglycosilated ovorubin determined by quadrupole ion trap nanoelectrospray MS/MS (ESI ToF/ToF). Inset: SDS-PAGE 8–20%. Lane 1: ovorubin; lane 2: MW markers; lane 3: Chemically-deglycosilated ovorubin. Bottom line, candidate sequence with homology to a Kunitz-type serine protease inhibitor.

Before mass spectrometry analysis, ovorubin was chemically deglycosylated. This treatment reduced subunit heterogeneity to a single 24 kDa band in SDS-PAGE (Figure 2, inset). Mass spectrometry analysis of the tryptic products of this band resulted in a well resolved fingerprint (Figure 2). The analysis showed one peptide with m/z = 1361.57 which matched (MASCOT score 71) the serine proteinase inhibitor from the insect *Sarcophaga bullata* (SBPI) (Figure 2). This protein belongs to the small Kunitz-type inhibitors family that features identically spaced cysteines, along a peptide chain of varying length [31].

Automated N-terminal Edman degradation identified 15 amino acid residues (Table 1). Interestingly, when the sequence was submitted to the NCBI non-redundant database without taxon restriction, no homology with known proteins was found.

**Table 1:** N-terminal amino acid sequence of deglycosilated ovorubin.

5	10	15
N K E X L	L L D I (I)	D A T T S

### Ovorubin – trypsin interaction

**Figure 3:**
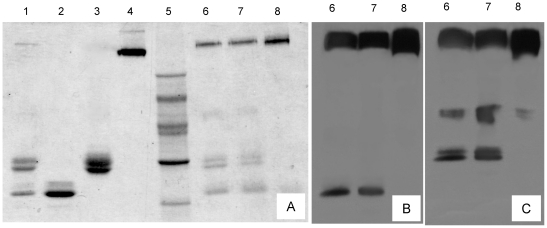
Analysis of ovorubin-trypsin cross-linked products by SDS-PAGE and immunobotting. (A): SDS-PAGE 8–20%. Lane 1: Ovorubin and trypsin mix; lane 2: Trypsin; lane 3: ovorubin; lane 4: cross linked ovorubin; lane 5: Molecular mass standards; lanes 6–8: ovorubin-trypsin mix +0.05, 0.20 and 0.80 mM DSP, respectively. (B): Western blot analysis of lanes 6, 7 and 8 using anti-trypsin antibody. (C): Western blot analysis of lanes 6, 7 and 8 using anti-ovorubin antibody.

Incubation of the digestive serine protease trypsin and ovorubin in the presence of the cross-linker dithiobis[succinimidyl propionate] (DSP) allowed the study of the interaction between both proteins. It should be noted that the cross-linking reaction is irreversible under the experimental conditions. As shown by SDS–PAGE (Figure 3), the cross-linked products, represented by the high molecular weight (MW) band, increase with increasing DSP levels all free trypsin being cross-linked at 0.8 mM DSP. (Figure 3A lanes 6, 7 and 8). This band was immunoreactive to both anti-trypsin and anti-ovorubin antibodies as shown by Western blot assay, thus confirming specific interaction between both proteins (Figure 3 B, C). The absence of ovorubin-ovorubin cross-linking was confirmed by subjecting purified ovorubin to DSP cross-linking. This treatment rendered a high MW band which showed a lower Rf value than the ovorubin-trypsin complexes (Figure 3, lane 4), and was not immunoreactive to anti-trypsin IgG antibody (data not shown).

The interaction was then further characterized by Small angle X-ray scattering (SAXS) experiments on the complex, providing an indication of its size. From the Guinier plots of free ovorubin and ovorubin-trypsin complex it was possible to fit a gyration radius of 40.10±0.80 Å and 44.05±1.20 Å, respectively. The gyration radii obtained for the ovorubin-trypsin complex are compatible with a 1∶1 stoichometry, whereas the gyration radii for free ovorubin are compatible with previous reports [28], that is, a compact oligomer of about 300 kDa, the MW determined for ovorubin [32].

### Trypsin inhibition

**Figure 4:**
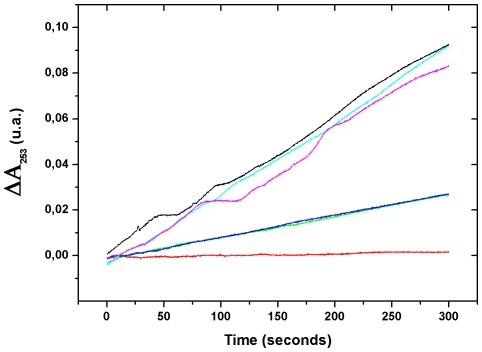
Effect of pH and temperature on trypsin inhibition capacity of ovorubin. Black line: negative control (no inhibitor); Red line: positive control (100% inhibition); Green line: pH = 7.4; Blue line: pH = 7.4+Ø; light blue line pH = 2.0; Violet line; pH = 2.0+Ø.

Trypsin inhibition properties of ovorubin were studied considering the effect of pH and temperature on this activity (Figure 4). The protein retained most of its inhibitory activity after heating at 100°C for 40 min at pH 7.4 (68.9±0.28% activity). In contrast, ovorubin lost almost all inhibitory activity by a combination of pre-incubation at pH = 2.0 for 48h followed by heating at 100°C for 40 min (3.4±0.07% activity) or by preincubation for 48 h at pH = 2.0 (3.0±0.20% activity).

### Antimicrobial activity of ovorubin

**Figure 5:**
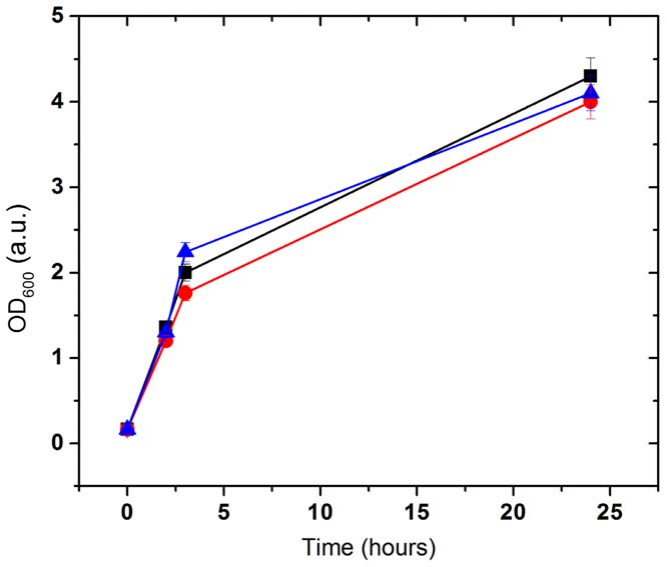
Effect of ovorubin on *E. coli* and *B. subtillis* growth. Bacteria were incubated in LB at 37°C, OD_600_ was measured at 2, 4 and 24 h. Black line: control; Red line: 100 µg ovorubin; Blue line: 20 µg ovorubin.

We tested the antimicrobial hypothesis adding ovorubin to bacterial cultures (*Escherichia coli* JM109, *Salmonella typhimurium*, *Bacillus subtilis* 168 and *Lactobacillus casei*) in liquid and solid media. Ovorubin showed no antibacterial activity against any of the strains tested, or the media employed in our experimental conditions (Figure 5).

### Simulated gastrointestinal digestion of ovorubin

The lack of antibacterial activity of ovorubin combined with a previous report indicating a high structural stability in a wide range of pH (pH 4.0–12.0) [28], suggested that the protein could be tailored to withstand the gastrointestinal tract of a predator. Therefore we tested this assumption *in vitro*, using a physiologically relevant digestion system, and then *in vivo*, by feeding studies (see below).

**Figure 6:**
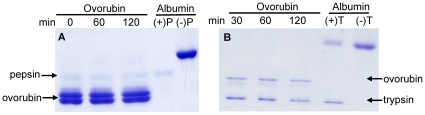
*In vitro* digestibility of ovorubin analyzed by SDS-PAGGE. (A) gastric digestion and (B) duodenal digestion. Lanes 1–3: 0, 60 and 120 min of incubation, lanes 4 and 5: positive (with enzyme) and negative control (without enzyme), respectively.

We found that ovorubin was resistant to simulated gastric digestion for 2 h, as shown by SDS-PAGE (Figure 6A). After this simulated gastric digestion, the pH was adjusted to duodenal conditions, trypsin was added and ovorubin simulated intestinal digestion performed for another 2 h. Again, ovorubin showed no significant alteration (Figure 6B).

### Effect of ovorubin-supplemented diet on rat growth rate

**Figure 7:**
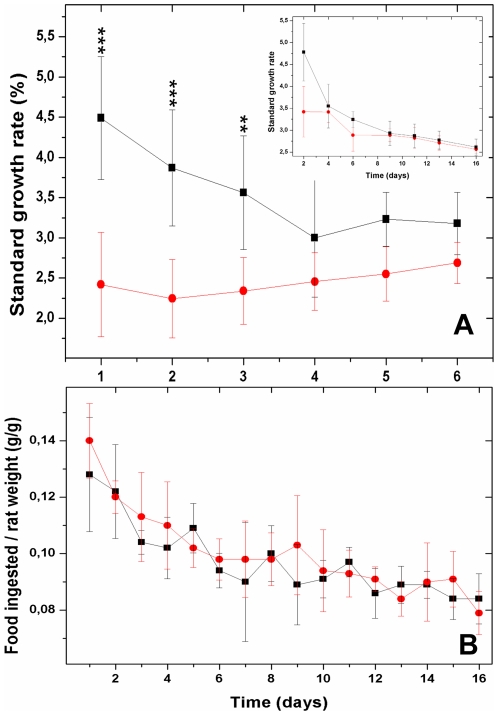
Effect of ovorubin supplemented diets on Wistar rats' standard growth rate and food consumption. (A) Standard growth rate during the first 6 days. Inset: Standard growth rate during 16 days showing rat adaptation to PI. Control (black square), treated (red circle). Values represent the mean ±1 SD (n = 12). *** *p*<0.001; ** *p*<0.01. (B) Food ingestion during a 16-day experiment.

Finally, a bioassay to test the biological effect of ovorubin was performed using rats. During the first 3 days of ovorubin oral administration the animals showed a significantly lower standard growth rate than the control ones (Figure 7A and inset). This effect on growth rate disappeared after the fourth day of treatment. Daily food ingestion was similar in control and ovorubin-supplemented rats along the experimental period (Figure 7B).

## Discussion

The principal functions so far attributed to egg proteins are: (i) provision of nutrients for the developing embryo; (ii) protection from microbial attack; and (iii) transport of nutrients into the developing embryo [33]. In the present study we provide evidence that ovorubin additionally functions as an antinutritive molecule, protecting the eggs against predation.

### Ovorubin is a small Kunitz-type proteinase inhibitor with many of the structural features of the family

Partial sequencing allowed the inclusion of ovorubin among the small Kunitz-type inhibitors family. This family includes many very well studied plant inhibitors, most of them with only one active site and resistant to proteolysis [34]; [35]. These characteristics are present in ovorubin, and provided us with the first clue on the role that its proteinase inhibitor function might play in the egg (see below). However, unlike most animal and plant proteinase inhibitors [36]–[38], ovorubin is a high MW oligomeric protein composed of several glycoforms and isoelectric point isoforms [27]. This large size for a single-site PI can be understood considering that ovorubin is in fact a multifunctional perivitellin displaying several other key functions related to the reproductive strategy of this freshwater snail [12]. Interestingly, the majority of Kunitz-type inhibitors are proteins with a molecular weight of about 20 kDa, which is the approximate MW of the deglycosilated ovorubin subunits (Figure 2) [27].

Despite its large size and oligomeric nature, ovorubin and the other members of the Kunitz-type inhibitors family share stability properties, such as a high structural stability in a pH range of at least 4.0–12.0 and a high thermostability [28]; [29]; [38]–[40]. This high structural stability was also reflected in its PI activity. Heating ovorubin at 100°C caused a minor loss to its trypsin inhibition capacity. PI activity was retained after a short exposure to acidic pH values (not shown), but was lost if exposed for 48 h at pH 2.0. This inactivation is in agreement with the unfolding and disassembling of the particle described for this protein at pH<4.0 [28]. Although proteinase inhibitors have been reported in eggs of several species, the use of SAXS and cross-linking provided the first demonstration, to our knowledge, of the protein-protein interaction of an egg proteinase inhibitor with a digestive protease in a stoichiometric relationship.

### The proteinase inhibitor role of ovorubin in eggs is not antimicrobial

Many serine proteinase inhibitors have been identified in egg-laying organisms such as arthropods, birds, and reptiles and invariably a role in either resistance to pathogens or the protection of critical peptides for embryo development has been ascribed to them [4]; [20]; [23]; [24]. In this line of thought a role to prevent microbial infection was assumed for ovorubin [24]. When we tested this assumption we found that ovorubin did not display antibacterial properties against Gram positive or Gram negative bacteria strains, at least in the experimental conditions used. In agreement with this lack of antimicrobial properties, a recent study reported that eggs of *P. canaliculata* can be experimentally infected by fungi [41]. Further, the absence of biochemical antimicrobial defenses in hard-shell eggs has been reported in other gastropods [2]; [16].

### Ovorubin PI activity is part of egg defense against predation

The PI functional features of ovorubin were not concurring with the roles classically ascribed to egg proteinase inhibitors. Surveying the literature on the roles of PIs we found that, like ovorubin, plant storage proteins in seeds [42], tubers [43] and fruits [44] have at the same time PI activity providing defenses against embryo predation. Moreover, these plant storage proteins/PIs share other biological activities with ovorubin. They are synthesized only in organs of reproduction, propagation and dispersal; they are accumulated in large amounts, display antioxidant properties and exhibit activities consistent with a role in protecting embryos from abiotic stresses [45]. However, in contrast to plant storage protein-PI, ovorubin has several additional protective functions as mentioned in the background section and discussed below [12]; [26]; [27].

Plant PIs in seeds and tubers comprise a complex defense system against insects, nematodes, birds and mammals by the inhibition of their digestive proteases, thus preventing the predator from digesting and incorporating nutrients from the tissues consumed [22]; [46]; [47]. Similarly, simulated gastrointestinal digestion showed that ovorubin withstands the harsh condition of the digestive tract. The high stability against pH of plant PIs is explained by the need of maintaining the native (active) conformation within the digestive fluids of predators [42]; [48]; [49]. In this regard, ovorubin pH stability falls within the pH range of vertebrate and invertebrate digestive tract fluids [50]–[53]. Thus, ovorubin could reach the predator's intestine in a fully active form as it has been reported for soybean Kunitz-type and other plant PIs fed to rats [54]. Feeding experiments provided *in vivo* evidence that ovorubin was indeed capable of decreasing rat growth rate during the first 3 days. The effect disappeared after continuous ovorubin feeding, probably because the animal adapts to the PI as it has been reported for several plant PI-herbivorous interactions [46]

Taking into account that trypsin has a highly conserved structure in animals, ovorubin inhibitory activity could be directed against the digestive tracts of a wide variety of organisms, including vertebrates [50]; [51]; [53] and insects [52], though this assumption needs experimental validation. Moreover, since trypsin catalytically activates the other gut protease zymogens, if this key enzyme of the cascade is blocked, it would render most of the other proteases also inactive. The action of the snail egg trypsin inhibitor on rats may therefore involve both the inhibition of trypsin activity (antidigestive role) and the resistance of the inhibitor to digestion by gut enzymes (antinutritive) limiting the predator's capacity to digest egg nutrients.

Though plant proteinase inhibitors have long been recognized as components in their defenses against predation, this is, to our knowledge, the first report in the animal kingdom.

### Ecological implications

Escaping predation is essential to survival for most animals and has resulted in the evolution of an amazing diversity of predator avoidance tactics. Among them, conspicuous coloration and unpalatability advertise chemical antipredator defense across many taxa. In this regard, there is a current debate regarding the allocation costs of avoiding predators: To effectively avoid predation, is it more advantageous to invest in increased conspicuousness or greater noxiousness, or to allocate equally to both signal modalities? [55]. In this study we present a novel alternative to the debate where there is no need of such trade-off, since noxiousness and conspicuousness are provided by the same molecule: ovorubin. In addition, by genetically encoding both the warning signal and the antinutritive/antidigestive defense, synthesis is even more cost-effective because females do not need to ingest toxic preys to endow eggs with chemical defenses. Furthermore, the “leftovers” of these defenses are in fact storage proteins consumed at a later time by developing embryos and hatchlings [5]. On the whole, apple snail egg defenses appears as a unique solution to allocation costs.

When considering the evolution of defenses, it is important to remember that something effective against one set of predators may be ineffectual against others. With only one reported predator worldwide, *P. canaliculata* eggs are an exception. It appears that their multifunctional perivitellins provide not only nutrients, but also a suite of defenses composed at least of antinutritive/antidigestive, neurotoxic and aposematic components (resumed in Table 2). These defenses acting simultaneously, and probably complemented by unpalatable factors, would impair the acquisition of nutrients and toxify the predator rendering *P. canaliculata* eggs unusually well defended. Regarding apple snail egg laying strategy to avoid predation it is important to note that there is neither ornamentation of the eggshell nor the use of external protection as oviposition on spiny vegetation or in protected areas (Figure 1, inset).

**Table 2:** Components of the biochemical defense system of *P. canaliculata* eggs.

Perivitellin	Composition	Feature	Role in defense	Reference
Ovorubin	Glyco-lipo-caroteno protein	Red-coloured	Aposematic (warning coloration)	[12]; [26]; [27]; [30]; [66]
Ovorubin	Glyco-lipo-caroteno protein	Proteinase inhibitor	Antinutritive/antitrypsin	Present paper
PV3	Lipo-caroteno protein	Orange-coloured	Aposematic	[12]; [32]
PV2	Glyco- lipoprotein	Lethal to mice	Neurotoxic	[13]; [14]; [30]; [66]

Considering that eggs with conspicuous coloration are very frequent across the Ampullariidae, this biochemical defense is probably not exclusive of *P. canaliculata*, and might be found more widely in other *Pomacea* with aerial oviposition, though more comparative work is needed to test this hypothesis.

Plant and apple snail embryos are sitting targets to predators, surrounded by highly nutritious compounds, and the evidence provided here suggests that both use proteinase inhibitors for protection. In plants, the loss of essential nutrients caused by these defensive proteins is predicted to be one of the most ecologically and evolutionally stable forms of defense against predation [56], this may very well be the case with apple snail eggs.

### Conclusions

This study shows that, in contrast with the classical assumption, ovorubin would not function as an antimicrobial agent in the eggs of *P. canaliculata*. Instead, we provide evidence for a different function of this proteinase inhibitor as part of the biochemical defenses of snail eggs against predation

Its structural and functional properties are similar to plant storage proteins that play a dual role to nourish embryos and as a defense against predators by limiting predator's ability to digest egg nutrients. This function for an egg proteinase inhibitor is to our knowledge, the first description in the animal kingdom.

Unlike plant proteinase inhibitors, ovorubin is actively involved in the defense of the embryos not only by rendering them antinutritive, but also by providing them with a genetically encoded warning signal, comprising a new level of coordination and complementation of egg defenses. This strategy is a novel alternative solution to energy allocation costs to avoid predation by combining toxicity and conspicuousness in the same molecule, opening new perspectives in the study of aposematism and mimicry.

The information gathered here and in previous reports indicates that the acquisition of this complex defense system including aposematic, neurotoxic and antinutritive components provides the eggs with a protection that predators have not managed to overcome yet. It is to our knowledge the first study that unveils the nature of the defenses of a prey which has virtually no predators.

Apple snail eggs provide an exceptional model to study the evolution of biochemical and physiological adaptations, which may have profound implications for addressing questions on ecology and evolution heretofore not fully appreciated.

## Methods

### Ethics Statement

All the studies performed with rats were approved by the Directive Board of the INIBIOLP and were carried out in accordance with the Guide for the Care and Use of Laboratory Animals [57]; (Instituto de Investigaciones Bioquimicas de La Plata's Animal Welfare Assurance No. A5647–01).

### Ovorubin isolation and purification

Adults of *P. canaliculata* were collected in streams or ponds near La Plata, province of Buenos Aires, Argentina. Eggs were collected from females either raised in our laboratory or taken from the wild between November and April (reproductive season). Embryo development was checked in each egg mass microscopically [32], and only egg masses having embryos developed to no more than the morula stage were used.

Methods for ovorubin purification have been described previously [25]. In short, egg homogenate was centrifuged sequentially at 10,000 xg for 30 min, and then at 100,000 xg for 60 min and the supernatant stored at −70°C until analysis.

The soluble protein fraction obtained was purified in a Merck-Hitachi high performance liquid chromatograph (HPLC) (Hitachi Ltd., Tokyo, Japan) by a serial HPLC purification method. First, the sample was analyzed in a Mono Q HR 10/10 (Amersham-Pharmacia, Uppsala, Sweden) using a gradient of 0–1 M NaCl in a 20 mM Tris buffer. The ovorubin peak was then further purified by size exclusion chromatography (Superdex 200 HR 10/20, Amersham-Pharmacia, Uppsala, Sweden) using an isocratic gradient of sodium phosphate buffer 50 mM, 150 mM NaCl, pH 7.6. Purity of the single peak obtained was checked by native PAGE performed in a Mini-Protean III System (Bio Rad Laboratories, Inc.) following manufacturer directions, MW standards were obtained from GE Healthcare (Uppsala, Sweden). Protein content was determined by the method of Bradford [58].

### Internal sequences determination by mass spectrometry

Ovorubin was first deglycosilated using trifluoromethansulfonic acid (TFMS, Sigma Chemical Co, St. Louis, USA) as described by Edge *et al*.[59] and the products were analyzed by SDS-PAGE. Peptide sequencing of tryptic digests of deglycosilated ovorubin was carried out by quadrupole ion trap nanoelectrospray MS/MS (ESI ToF/ToF) in an LCQ instrument (Finnigan TermoQuest, San Jose, CA), at the Proteomic Service, National Centre of Biotechnology, Madrid, Spain. The interpretation of MS/MS spectra was done manually, but assisted by various software packages, including Mascot (Matrix Science Ltd., London) and MSProduct, a facility of the Protein Prospector package [60].

### N-Terminal amino acid sequence determination

Sequencing was performed by automatic Edman degradation at Laboratorio Nacional de Investigación y Servicios en Péptidos y Proteínas (LANAIS-PRO, Universidad de Buenos Aires - CONICET). The system used was an Applied Biosystems 477A Protein/Peptide Sequencer interfaced with a 120 HPLC for one-line phenylthiohydantoin amino acid analysis.

### Trypsin inhibition assays

In order to test the effect of pH and temperature on ovorubin trypsin inhibition, ovorubin solutions (0.5 mg/ml) at pH 2.0 and 7.0 were heated at 100°C for 40 min. After this treatment, ovorubin preparations were incubated with a 10 fold molar excess of trypsin for 1 h and trypsin inhibition determined [61]. In short, N-benzoil-L-arginine ethyl ester (BAEE) is hydrolyzed by trypsin at the ester linkage causing an increase in absorbance at 253 nm at 25°C. Results were expressed as units of activity (the amount of enzyme that causes an absorbance increase of 0.003 per minute at 25°C).

### Interaction between ovorubin and trypsin

The interaction was analyzed by cross-linking experiments as well as by small angle X-ray scattering (SAXS).

For the *in vitro* chemical cross-linking, purified ovorubin (5mg/ml) and trypsin (5 mg/ml) (Sigma) in a total volume of 200 µl were cross-linked for 30 min at room temperature using DSP (Pierce, IL, USA) at final concentrations of 0.05, 0.2 and 0.8 mM in a reaction buffer composed of 0.1M phosphate, 0.15M NaCl, pH 7.2. Ovorubin self cross-linking was checked at 0.8 mM DSP. Reaction was terminated by the addition of 1.0 M Tris, pH 7.5 to a final concentration of 50 mM. The complexes were analyzed by 8–20% SDS-PAGE, transferred onto nitrocellulose membranes and subjected to immunoblotting, as described previously [62]. For the complex detection, membranes were incubated for 2 h with an anti-trypsin polyclonal antibody (Santa Cruz Biotecnology, Inc.) (diluted 1∶5,000) and an anti-ovorubin polyclonal antibody in 10 mM Tris-HCl, pH 7.4, 0.15 M NaCl. Specific antigens were detected by goat anti-rabbit IgG horseradish peroxidase conjugate (Bio-Rad Laboratories) diluted (1∶3,000). Immunoreactivity was visualized by electrochemiluminescence.

The interaction between ovorubin and trypsin was also studied by SAXS experiments. Complexes obtained by chemical cross-linking were purified by size exclusion chromatography and purity checked by native electrophoresis, as described in the purification section. Experiments were performed at the D02A-SAXS2 line operating in the Laboratório Nacional de Luz Síncrotron, Campinas (SP, Brazil). The scattering pattern was detected using a MARCCD bidimensional charge-coupled device assisted by FIT2D v12.012 software [63]. The experiments were performed using a wavelength of 1.448 Å for the incident X-ray beam to minimize carbon absorption. The distance between the sample and the detector was kept at 1044 mm, allowing a Q-range between 0.012 and 0.25 Å^−1^ (nominal D_max_ ≤260 Å). The temperature was controlled using a circulating water bath, and kept at 25°C. Each individual run was corrected for sample absorption, photon flux, buffer scattering, and detector homogeneity. At least three independent curves were averaged for each single experiment. SAXS experiments in a protein range of 2.4–0.20 mg/mL were performed to rule out a concentration effect in the data. The size of ovorubin-trypsin complex was determined using the gyration radii (*R*_G_) obtained by analysis of SAXS patterns as Guinier plots (ln(I) = ln(I_0_)−*R*_G_Q^2^/3, Q = 4πsin(θ)/λ, *R*_G_Q≤1).

### Antimicrobial Activity Assays

The antimicrobial activity of ovorubin was tested on Gram (+) (*E. coli* JM109 and *S. typhimurium*) and Gram (-) strains (*B. subtilis* 168 and *L. casei*), both in solid and liquid media. For all the tests the microorganisms were grown overnight to mid-logarithmic phase in Luria-Bertani broth (LB) for *E. coli*, *S. typhimurium* and *B. subtilis* and de Man, Rogosa and Sharpe (MRS) broth for *L. casei*. For the solid medium assay, 50 µl of each culture were spread onto LB/agar or RMS/agar plates, and 20 min later 10 µl drops containing 20 µg, 10 µg and 2 µg of ovorubin were dispensed on each plate; sterile phosphate buffer was used as negative control. The plates were incubated for 18 h at 37°C and the formation of inhibition rings was observed. The liquid media assays were performed using one *E. coli* (JM109) and *B. subtilis* (168) strains, grown as indicated above. Aliquots of culture were diluted with fresh medium in glass test tubes to obtain an OD_600_ = 0.19, and supplemented with 100, 20, 10, or 2 µg of ovorubin, respectively; sterile buffer was used as control. The tubes were then incubated at 37°C with vigorous shaking and changes in OD_600_ recorded.

### In vitro ovorubin digestibility

The simulated gastrointestinal digestion of ovorubin was performed *in vitro* following the method previously described by Moreno [64] with slight modifications. Briefly, gastric digestion was performed at 37°C for 120 min at pH 2.5 in the presence of porcine pepsin (Sigma, Dorset, UK; product No. P 6887) at a ratio of enzyme: substrate 1∶20 (w/w). Aliquots were taken at 0, 60 and 120 min and analyzed by SDS-PAGE as described above. The digestion was stopped by raising the pH to 7.5 using 50 mM phosphate buffer. For *in vitro* duodenal digestion the 120 min gastric digest was used as starting material. The duodenal digestion was performed using trypsin from bovine pancreas (Sigma, product No. T 9935) at a ratio of enzyme: substrate 1∶400 (w/w), at 37°C taking aliquotes at 0, 60 and 120 min for SDS-PAGE analysis. Albumin was used as positive (with enzyme) and negative control (without enzyme) in both gastric and duodenal digestion.

### Effect of ovorubin supplemented diet on rat growth

Male Wistar rats 6 weeks old (weighing approximately 180 g at the start of the experiments) were separated into two groups (control and treated) of 12 animals each and fed *ad libitum* with a commercial diet for 16 days. The treated group was orally administered 100 µl of purified ovorubin (4 mg/ml) in 50mM phosphate buffer pH 7.4 on a daily basis, while the control group received 100 µl of buffer. Food consumption as well as body weight was determined daily for each animal. The standard growth rate (SGR) was calculated as follows:



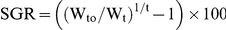



Where W_to_ is the initial weight, W_t_ is the final weight, and t is the time in days [65].

The experiment was replicated twice. Data were analyzed by one-way ANOVA using Instat, v. 2.0 (GraphPad, San Diego, CA) and considered significant at a level of 5%.
